# Validation of the emergency surgery score’s predictive accuracy for postoperative outcomes and ICU admissions in MENA vs. non-MENA emergency surgery patients

**DOI:** 10.1007/s00068-025-02888-6

**Published:** 2025-05-22

**Authors:** Mohamed Said Ghali, Samer A. Hasan, Ahmad R. Al-Qudimat, Mohammed Alabidi, Omar S. Moustafa, Raed M. Al-Zoubi

**Affiliations:** 1 Department of Acute Care Surgery, Hamad Medical Corporation, Doha, Qatar; 2 Department of General Surgery, Ain Shams University, Cairo, Egypt; 3 Weill Cornell Medicine-Qatar, Doha, Qatar; 4 Public Department, QU-Health, College of Health Sciences, Qatar University, Doha, Qatar; 5 Surgical Research Section, Department of Surgery, Hamad Medical Corporation, Doha, Qatar; 6 Department of Biomedical Sciences, QU-Health, College of Health Sciences, Qatar University, 2713 Doha, Qatar; 7 Department of Chemistry, Jordan University of Science and Technology, P.O.Box 3030, Irbid, 22110 Jordan

**Keywords:** Emergency laparotomy, Emergency surgery score, Validation, MENA, Predictor

## Abstract

**Background:**

The Emergency Surgery Score (ESS) has demonstrated strong predictive value for morbidity, mortality, and long-term survival outcomes. However, its applicability and validity in the Middle East and North Africa (MENA) region remain understudied. This research seeks to validate ESS's ability to predict postoperative outcomes, including 30-day mortality, complications, and ICU admissions, among patients undergoing emergency laparotomies (EL).

**Methods:**

This retrospective study analyzed 230 EL cases from 2017 to 2021. ESS scores were calculated for each patient, and its predictive accuracy was compared with the American Society of Anesthesiologists (ASA) classification using c-statistic methodology. We also compared postoperative outcomes between MENA and non-MENA cohorts to assess potential regional variations in ESS performance.

**Results:**

Out of 230 patients, 118 were from MENA and 112 from non-MENA regions. Sepsis was the most common diagnosis (69.6%). ICU admission was recorded in 63.4% of cases, and the 30-day mortality rate was 13.91%. ESS scores did not differ significantly between MENA and non-MENA patients (median: 7.5 vs. 7; *P* = 0.45). ESS outperformed ASA in predicting postoperative outcomes: complications (c-statistic: 0.79 vs. 0.73), ICU admissions (0.81 vs. 0.76), and mortality (0.86 vs. 0.78). Optimal ESS cutoffs for complications, ICU need, and mortality were 6, 8, and 10, respectively. ESS performed similarly across both MENA and non-MENA populations in predicting mortality, complications, and ICU admissions.

**Conclusion:**

The ESS is a superior tool compared to ASA for predicting postoperative outcomes in emergency surgical patients, and it is applicable to diverse populations, including those from the MENA region. ESS enhances preoperative risk stratification, informs counseling decisions, and supports quality benchmarking across different healthcare settings. Future studies should address potential biases, including selection and information bias, and further explore ESS's role in different cultural contexts.

## Introduction

Emergency general surgery (EGS) imposes a substantial and steadily rising burden on the healthcare system due to its associated increases in mortality and morbidity [1]. Emergency laparotomy (EL), a critical EGS procedure, is performed on patients with a high-risk condition for a range of underlying emergency surgical diseases [2]. The mortality rates following emergency laparotomy vary significantly, and there is a need for accurate risk assessment tools to predict outcomes and guide clinical decision-making [3]. The Emergency Surgery Score (ESS) has emerged as a promising tool for evaluating risk in EL patients. Developed from a large contemporary dataset of patients undergoing EL, ESS has been validated in several populations, demonstrating its potential for improving patient outcomes and informing clinical management [3, 4].

Given the complexity of emergency surgeries, the role of validated risk assessment tools like ESS becomes crucial in standardizing the evaluation and prediction of outcomes for EL patients. This is especially important considering the heterogeneous nature of patients undergoing EL, who are often presented with a wide range of underlying conditions. ESS may help standardize the approach to evaluating these patients, potentially leading to more consistent and improved outcomes across different populations [2, 5, 6]. This is particularly critical in light of the high morbidity and mortality associated with EL, as well as the need for efficient risk assessment tools to guide clinical care, enhance patient survival, and inform preoperative counseling for both patients and their families [7].

In the Middle East and North Africa (MENA) region, patients often seek information about the risks of surgery, including the likelihood of complications and success rates. Similar concerns are observed in non-MENA patients—often immigrants—who may be anxious about receiving different levels of care compared to citizens of the host country. Given the diverse and multiethnic nature of the MENA population, there is a need for a reliable and standardized tool like ESS to address these concerns and provide effective preoperative counseling. While the American Society of Anesthesiologists (ASA) score is currently used at our institution to assess patient risk, it does not fully address postoperative complications or the need for intensive care unit admissions. Therefore, we aim to retrospectively validate the performance of ESS in predicting postoperative outcomes for EL patients at our institution in Qatar, where we provide care for patients with acute surgical conditions. The effectiveness of ESS has been demonstrated in various countries, making it a valuable tool to evaluate its performance in our population.

## Methodology

### Study design and population

This retrospective study included all patients aged 18 years and above who underwent emergency laparotomy (EL) procedures at Hamad Medical Corporation, the primary healthcare facility in Qatar, between January 2017 and December 2021. EL was operationally defined as any surgical abdominal intervention performed promptly following the patient's diagnosis, where any delay could potentially compromise the patient's health and prognosis. Exclusions were made for emergent procedures related to trauma, vascular issues, and gynecological conditions, as well as elective laparotomies, laparoscopic surgeries, hernia repairs, and soft tissue procedures. Patients lacking the necessary data to calculate the Emergency Surgery Score (ESS) were excluded from the analysis.

### Calculating ESS

ESS was calculated for each patient using pre-operative demographic variables (age, race, BMI, and transfer from an external inpatient facility or emergency department), comorbidities (ascites, hypertension [HTN], chronic obstructive pulmonary disease [COPD], dyspnea, disseminated cancer, functional dependency, steroid use, ventilator requirement within 48 h preoperatively, and weight loss > 10% in the preceding 6 months), and laboratory values (albumin, alkaline phosphatase, BUN, creatinine [Cr], INR, platelet count [PTL], AST, sodium [Na], and WBC). Each variable was assigned a point value based on its contribution to the overall risk score for patients undergoing emergency surgery (Table 1).

**Table 1:** Emergency surgery score and component variables [4]

Variable	Points
Age > 60 years	2
Albumin < 3.0 u/l	1
Ascites	1
Body mass index < 20 kg/m2	1
BUN > 40 mg/Dl	1
History of COPD	1
Disseminated cancer	3
Dyspnea	1
Functional dependence	1
Hypertension	1
INR > 1.5	1
Platelets < 150 *10^3	1
SGOT > 40 U/L	1
Sodium > 145 mg/dL	1
Steroid use	1
Transfer from outside ED or acute care hospital	1
Ventilator requirement 48 h pre-operativel	3
WBC < 4.5 X 103/µL or 15–25 X 103/µL	1
WBC > 25 X 103/µL	2
White race	1
> 10% weight loss in last 6 months	1
Total	29

### Study outcomes

The primary outcome of the study was 30-day mortality, while secondary outcomes included 30-day postoperative morbidity and the requirement for ICU admission. Definitions were based on those provided by the American College of Surgeons National Surgical Quality Improvement Program (ACS-NSQIP). Specifically:

- **30-day mortality** referred to the death of a patient within 30 days following the index emergency laparotomy.
- **Morbidity** encompassed various complications, including superficial surgical site infection (SSI), deep SSI, organ/space SSI, wound dehiscence, pneumonia, unplanned intubation, pulmonary embolism, failure to wean off ventilator for more than 48 h postoperatively, progressive renal insufficiency, acute kidney injury, urinary tract infection, stroke (CVA), cardiac arrest requiring cardiopulmonary resuscitation, myocardial infarction, bleeding necessitating transfusion, deep venous thrombosis, sepsis, and septic shock.
- **ICU admission** was defined as the need for ICU-level care at any point during the index hospitalization.

We compared the predictive power of ESS with the American Society of Anesthesiologists (ASA) classification, the main assessment tool currently used at our institution. Additionally, we compared the study variables and ESS’s prediction for postoperative complications, mortality, and the need for ICU admissions between MENA and non-MENA patient populations.

### Ethical considerations

Prior to data collection, approval for the study was obtained from the Institutional Review Board at Hamad Medical Corporation (approval No. **MRC-01–23–615**).

### Statistical analysis

All statistical analyses were performed using STATA v17. Descriptive statistics summarized the baseline characteristics, while chi-square tests and logistic regression were used to evaluate associations between variables. The predictive capacity of ESS for both 30-day mortality and complications was assessed using logistic regression modeling, with odds ratios (ORs) and 95% confidence intervals (CIs) reported.

The predictive performance of ESS was evaluated using Receiver Operating Characteristic (ROC) curve analysis, facilitating the computation of the c-statistic (Area Under the Curve, AUC), a well-established measure of model discrimination. The AUC ranges from 0.5 (no discriminative ability) to 1 (perfect discrimination). An AUC score between 0.7 and 0.8 is considered acceptable, 0.8 to 0.9 is excellent, and above 0.9 is outstanding. ESS’s predictive performance was compared with the ASA classification by juxtaposing their respective AUC scores, with statistical tests (e.g., DeLong's test) used to evaluate the significance of differences. Additionally, a subset analysis explored ESS’s predictive capacity for specific complications, providing a more granular assessment of its utility in predicting adverse outcomes in surgical settings.

## Results

### Patient characteristics

Among our 230 patients (118 from MENA and 112 from Non-MENA) who underwent EL procedure and matched our inclusion criteria, 156 were male, accounting for 67.8% of the study population, with a male-to-female ratio of ≃2:1. We correlated study variables based on patient gender, we found that female had higher BMI (*P* = 0.001), female patients (62.16%) were more in MENA countries, more female had functional dependence before surgery (*P* = 001), more female had hypertension (*P* = 0.004), diabetes mellitus (*P* = 0.22), and more female present with tachycardia to emergency (*P* = 0.019). conversely, male patients (53.8%) were more prevalent in non-MENA population and had higher serum ALP up on admission than female gender (*P* = 0.008). rest of correlation between male and female gender are insignificant.

### MENA vs non-MENA

**Fig. 1:**
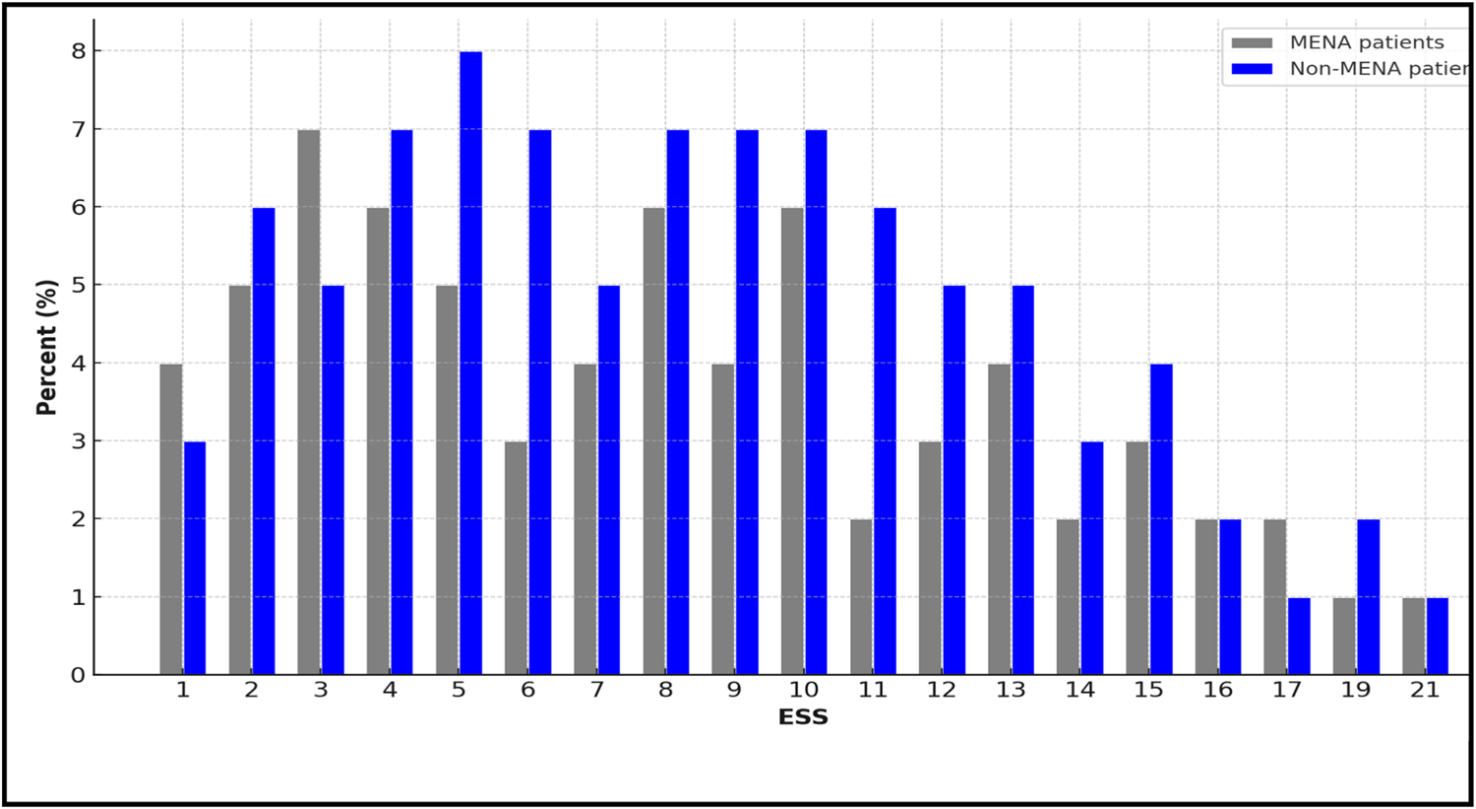
Bar graph showing the percentage distribution of ESS values between patients from MENA (gray bars) and non-MENA (blue bars) populations

The study compared the ESS and other variables between MENA and non-MENA patients was displayed in Fig. 1. The mean age of the MENA group was significantly higher (57.94 ± 18.33) than the non-MENA group (46.55 ± 12.20), with a *P*-value of < 0.001. Similarly, the MENA group exhibited a higher mean BMI (28.9 ± 6.1) compared to the non-MENA group (24.6 ± 4.7), also statistically significant (*P* < 0.001). Gender distribution showed notable differences, with the non-MENA population having a higher proportion of males (75% vs. 61% in MENA), reaching significance (*P* = 0.023). Vital signs such as systolic blood pressure, temperature, respiratory rate, and heart rate were comparable between the two groups, showing no statistically significant differences. In terms of comorbidities, statistically significant differences were observed in categories such as BMI < 20 kg/m^2^ (*P* = 0.046), disseminated cancer (*P* = 0.043), functional dependency (*P* < 0.001), history of COPD (*P* = 0.049), and hypertension (*P* < 0.001), with higher rates in the MENA group. The remaining comorbidity factors, including ventilator requirement and weight loss, did not show significant differences. Laboratory values, such as albumin, BUN, creatinine, and INR, were not significantly different between the two groups. However, the ASA score was higher in the MENA group (3.08 ± 0.95 vs. 2.79 ± 0.93, *P* = 0.024), indicating a slightly greater preoperative risk. The ESS score, however, did not differ significantly between the populations (median [IQR] 7.5 [5,12] for MENA vs. 7 [5, 10] for Non-MENA, *P* = 0.45) (Table 2).

**Table 2:** Comparison of demographics, comorbidities, laboratory findings, and clinical characteristics between MENA and Non-MENA patients undergoing ESS

Variable	MENA *N* = 118	Non-MENA *N* = 112	Total *N* = 230	*P*-Value
Age (Mean ± SD)	57.94 (18.33)	46.55 (12.20)	52.40 (16.62)	< 0.001*
BMI (Mean ± SD)	28.9 (6.1)	24.6 (4.7)	26.8 (5.9)	< 0.001*
Gender				0.023*
Male	72 (61.0%)	84 (75.0%)	156 (67.8%)	
Female	46 (39.0%)	28 (25.0%)	74 (32.2%)	
Vital signs				
Systolic BP > 90	111(94.1%)	100(89.3%)	211(91.7%)	0.19
Temperature > 38	7(5.9%)	7(6.25%)	14(6%)	0.58
Respiratory rate > 21	42(35%)	37(33%)	79(33.3%)	0.82
Heart rate > 100	60(50.8%)	49(43.8%)	109(47.4%)	0.28
Comorbidities				
BMI < 20 kg/m2	6 (5.1%)	14 (12.5%)	20 (8.7%)	0.046*
Ascites	85 (72.0%)	83 (74.1%)	168 (73.0%)	0.72
Disseminated cancer	12 (10.2%)	22 (19.6%)	34 (14.8%)	0.043*
Dyspnea	54 (45.8%)	53 (47.3%)	107 (46.5%)	0.81
Functional dependency	29 (24.6%)	7 (6.3%)	36 (15.7%)	< 0.001*
History of COPD	4 (3.4%)	0 (0.0%)	4 (1.7%)	0.049*
Hypertension	53 (44.9%)	26 (23.2%)	79 (34.3%)	< 0.001*
S teroid use	13 (11.0%)	7 (6.3%)	20 (8.7%)	0.20
Ventilator requirement within 48 h preoperatively	11 (9.3%)	7 (6.3%)	18 (7.8%)	0.63
Weight loss > 10% in the preceding 6 months	4 (3.4%)	9 (8.0%)	13 (5.7%)	0.13
Laboratory findings				
Albumin < 3.0 U/L	74 (62.7%)	60 (53.6%)	134 (58.3%)	0.16
BUN > 40 mg/dL	109 (92.4%)	102 (91.1%)	211 (91.7%)	0.72
Creatinine > 106.1umol or > 1.2 mg/Dl	44 (37.3%)	37 (33.0%)	81 (35.2%)	0.50
INR > 1.5	24 (20.3%)	26 (23.2%)	50 (21.7%)	0.60
Platelets < 150 × 103/μL	21 (17.8%)	22 (19.6%)	43 (18.7%)	0.72
WBC, × 103/μL > 25	6 (5.1%)	12 (10.7%)	18 (7.8%)	0.11
Sodium > 145 mg/dL	15 (12.7%)	6 (5.4%)	21 (9.1%)	0.053
ASA score (Mean ± SD)	3.08(0.95)	2.79(0.93)	2.94(0.95)	0.024*
ESS score (Median (IQR))	7.5(5,12)	7(5,10)	7.0(5,11)	0.45

*BMI* body mass index, *TIA* transient ischemic attack, *BP* blood pressure, *WBC* white blood cell, *INR* international normalized ratio, *BUN* blood urea nitrogen, *AST* aspartate aminotransferase, *ALP* alkaline phosphatase. Values are presented as number (%), mean ± standard deviation, or median (interquartile range) as appropriate. Statistically significant p-values are indicated with an asterisk ().*

Our cohort of 230 patients (118 from MENA and 112 from non-MENA regions) presented with diverse etiologies necessitating emergency laparotomy. Hollow viscus perforation was the most prevalent indication (*n* = 83, 36.1%), followed by intestinal obstruction (*n* = 72, 31.3%). Other significant etiologies included mesenteric ischemia (*n* = 28, 12.2%), anastomotic leaks/collections (*n* = 20, 8.7%), and gastrointestinal bleeding (*n* = 16, 7.0%). Complicated acute appendicitis represented the least common indication (*n* = 11, 4.8%). This distribution demonstrates that hollow viscus perforation and intestinal obstruction collectively accounted for two-thirds of cases, reflecting the predominant surgical emergency patterns in our study population.

Table 3 summarizes the incidence of postoperative complications following emergency surgery. Sepsis emerged as the most frequent complication, occurring in 69.6% of cases. In contrast, graft/prosthesis/flap failure represented the rarest complication, observed in only one patient (0.4% of patients).

**Table 3:** Incidence of postoperative complications following ESS

Complication	Number, (%)
Superficial surgical site infection	87(37.8)
Deep surgical site infection	60(26.1)
Organ/space surgical site infection	57(24.8)
Abdominal wall dehiscence	26(11.3)
Anastomotic leak	21(9.1)
Bowel diversion	74(32.2)
Progression of baseline renal insufficiency with creatinine level of greater than 176.8 umol/L	52(22.6)
Urinary tract infection	14(6.1)
Transfusion-requiring hemorrhage	68(29.6)
Graft/prosthesis/flap failure	1(0.4)
Pneumonia	23(10)
Unplanned intubation	30(13)
Pulmonary embolism	3(1.3)
Failure to wean off ventilator 48 h after surgery	70(30.4)
Acute kidney injury requiring dialysis	26(11.3)
Cerebrovascular accident neurological deficit	4(1.7)
Cardiac arrest requiring cardiopulmonary resuscitation	28(12.2)
Myocardial infarction	8(3.5)
Deep vein thrombosis	3(1.3)
Sepsis	160(69.6)
Septic shock	100(43.3)

The median duration of hospital stay was 14 days (interquartile range: 8–33 days). A total of 63.4% of patients required ICU admission, and the 30-day mortality rate was 13.91% (32 patients) (Table 4).

**Table 4:** ICU admission, 30-day mortality, and hospital length of stay

Variable	Number (%), Median (IQR)
ICU admission	145(63.04)
30-day of mortality	32(13.91)
length of hospital stays (LOS) in days	14(8–33)

### Validation of ESS as an outcome predictor

#### ESS & post-operative complication’s rate

Figure 1-A illustrates the distribution of preoperative complications across ESS scores. Complications were predominantly observed in patients with ESS scores ranging from 4 to 11, with peak incidence occurring at score 10. This bimodal distribution suggests that patients with moderate ESS scores have the highest risk of developing complications, while those at both extremes of the scoring spectrum (very low or very high scores) demonstrated lower complication rates.

**Fig. 2:**
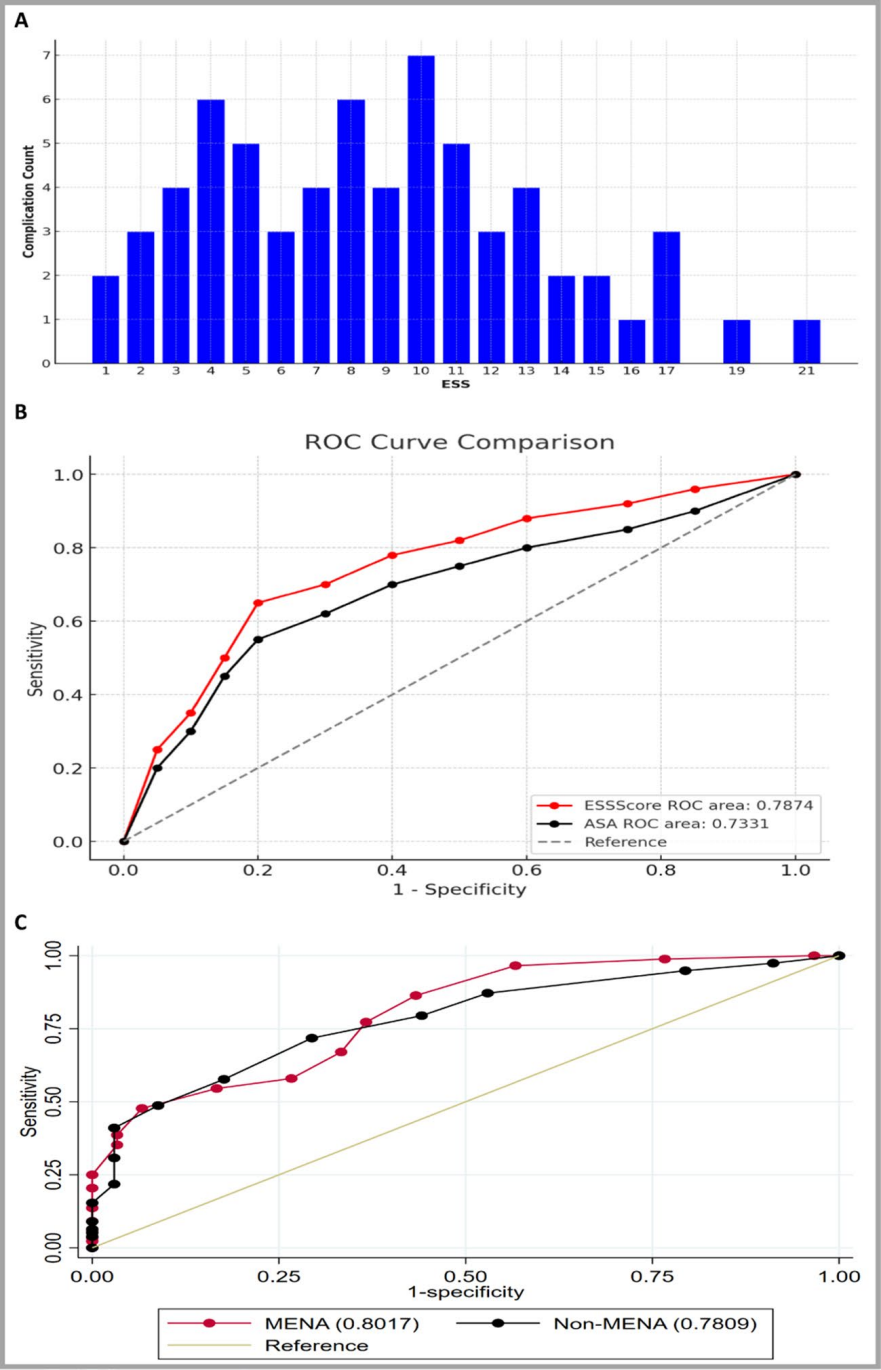
Receiver operating characteristics (ROC) curve analysis. (**A**) Distribution of Complications per ESS score. (**B**) Comparison of ROC curves using ESS score and ASA score to complications in predicting complications, with reference line. (**C**) ROC curve comparison for ESS score between MENA vs non-MENA

ROC curve indicates that higher ESS scores are strongly associated with increased complications rates, with a critical threshold at scores of 10 and above, where the risk of complications significantly rises. ESS and ASA were significant predictors of post operative complications with more association toward ESS score, with a c-statistic of 0.79 (95% CI = 0.72–0.84), and a c-statistic of 0.7331(95% CI: 0.66–0.79) respectively (Fig. 2B).

The ROC curve compares the predictive performance of the ESS score in identifying major complications for MENA and non-MENA populations. The ROC area for MENA (AUC = 0.8017) suggests good discriminative ability, slightly outperforming the non-MENA group (AUC = 0.7809). Both curves demonstrate that the ESS score is a reasonably effective tool for predicting complications, as indicated by the upward trend and areas under the curve being above 0.75 for both groups. The optimal cutoff score for predicting complications was determined to be 6 (Fig. 2C).

We performed a subset analysis to evaluate the predictive capacity of ESS in anticipating specific complications. Table 4 outlines the effectiveness of ESS in predicting some prevalent complications, as indicated by the c-statistic. Specifically, ESS demonstrated robust predictive capabilities for myocardial infarction, with a c-statistic of 0.92 (95% CI: 0.85–0.99), acute kidney injury necessitating dialysis, with a c-statistic of 0.88 (95% CI: 0.83–0.94), and cardiac arrest requiring cardiopulmonary resuscitation, with a c-statistic of 0.88 (95% CI: 0.83–0.93) (Table 5).

**Table 5:** ESS a predictor of other complications

Complications	C-statistics (95% CI)
Sepsis	0.74(0.67–0.81)
Superficial surgical site infection	0.55(0.48–0.63)
Organ space surgical site infection	0.63(0.55–0.70)
Pulmonary embolism	0.60(0.45–0.744)
Failure to wean off intubator 48 h after surgery	0.79(0.37–0.86)
Acute kidney injury requires dialysis	0.88(0.83–0.94)
Cardiac arrest requires cardiopulmonary resuscitation	0.88(0.83–0.93)
Myocardial infarction	0.92(0.85–0.99)

### ESS as an ICU admission’s predictor

**Fig. 3:**
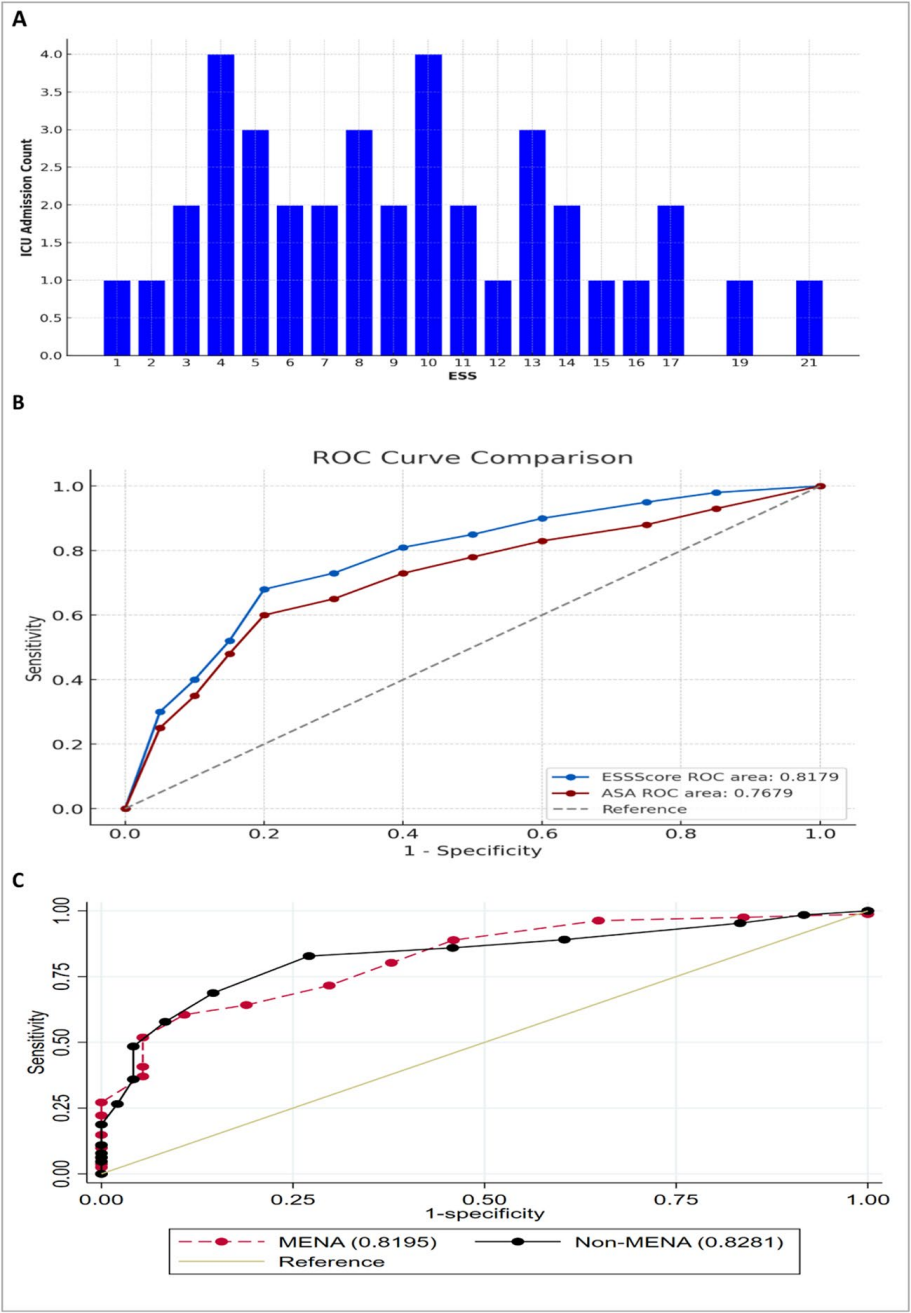
**A** Distribution of ICU admission to ESS score. (**B**) ROC curves for ESS and ASA score for ICU admission. (**C**) ROC curve comparison of ESS score for predicting ICU admission between MENA and Non-MENA populations

The distribution of ICU admission as per ESS, highest ICU admissions are observed around ESS 4 and 10, indicating that patients within these score ranges are more likely to be admitted to the ICU. The distribution of ICU admissions is relatively consistent across mid-range ESS scores, with fewer admissions seen at the extremes (ESS scores 1, 2, and 21) (Fig. 3A).

The ROC curve (Fig. 3B) indicates that higher ESS scores are strongly associated with increased ICU admission rates, with a critical threshold at scores of 10 and above, where the risk of ICU admissions significantly rises. ESS and ASA were significant predictors of ICU admission with higher prediction toward ESS, with a c-statistic of 0.81 (95% CI: 0.76–0.87), and c-statistic of 0.76(95% CI: 0.70–0.82).

In the ROC curve, the area under the curve (AUC) for both groups indicates good predictive power, with MENA having an AUC of 0.8195 and non-MENA slightly higher at 0.8281. This suggests that the ESS score is similarly effective in predicting ICU admissions for both MENA and non-MENA populations, The optimal cutoff score for predicting complications was determined to be 8 (Fig. 3C). Finally, the non-MENA population shows a marginally better discriminative ability in identifying patients at risk of ICU admission.

### ESS as a 30-day mortality predictor

**Fig. 4:**
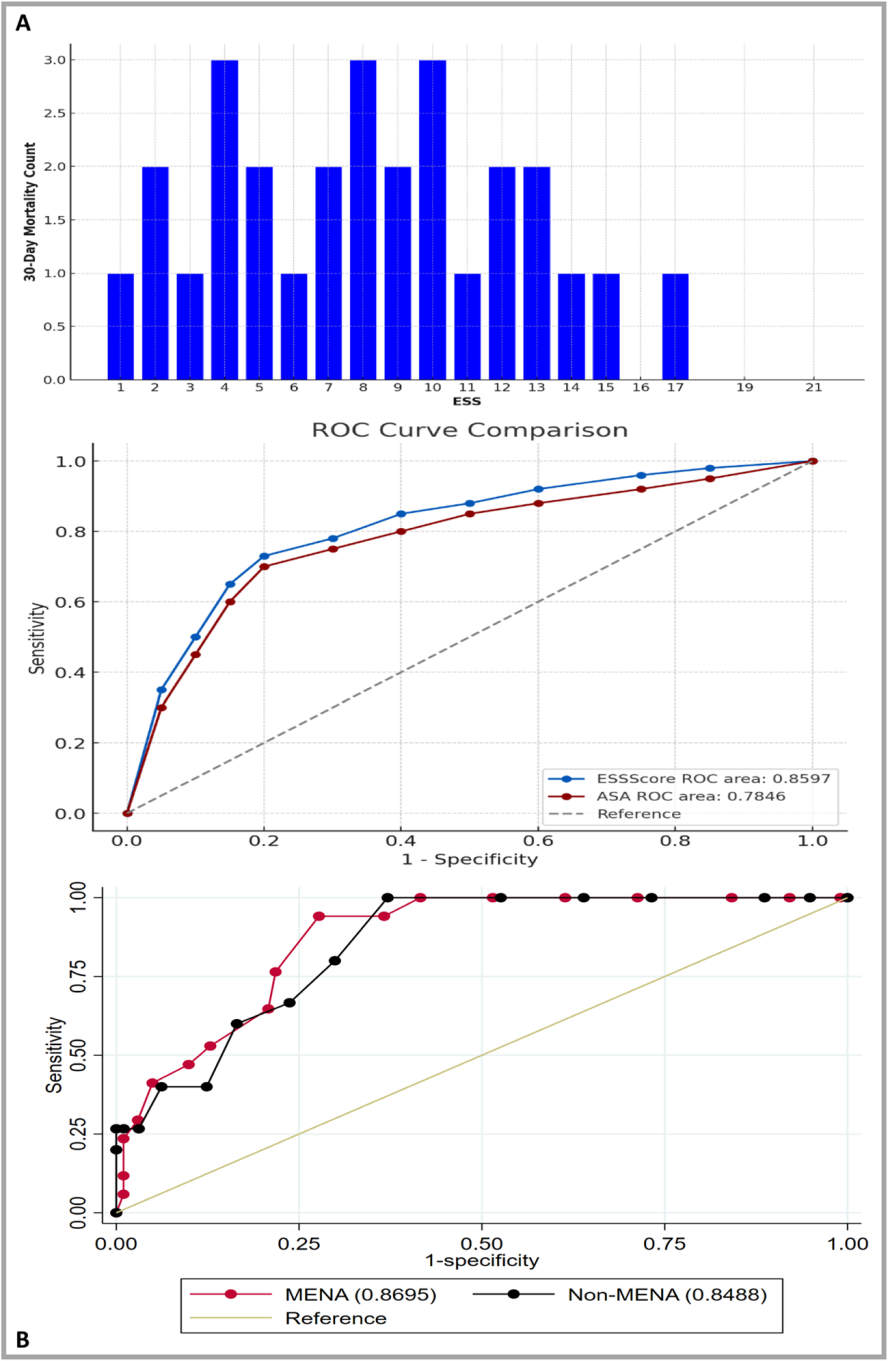
**A** Distribution of 30-day mortality to ESS score. (**B**) ROC curves comparing ESS and ASA score for 30-day mortality prediction. (**C**) ROC curve comparison of ESS score for predicting 30-day mortality between MENA and Non-MENA populations

Figure 4A shows the frequency of the 30 day of mortality per ESS, highest mortality counts are seen at ESS 4 and 10, with consistent mortality occurring across a broad range of mid-level ESS scores. This suggests that patients with moderate ESS scores, particularly around 4 and 10, are more likely to experience 30-day mortality, while those at the extremes of the ESS scale tend to have lower mortality rates.

This trend indicates that higher ESS scores are strongly associated with increased 30-day mortality rates, with a critical threshold at scores of 10 and above, where the risk of mortality significantly rises. ESS and ASA were significant predictors of 30-day mortality but also ESS was more significant with a c-statistic of 0.86 (95% CI: 0.80–0.91), and a c-statistic of 0.78 (0.71–0.85) (Fig. 4B).

AUC for MENA is 0.8695, indicating strong discriminative ability, while non-MENA has a slightly lower AUC of 0.8488. Both populations exhibit good predictive performance, with the ESS score proving to be a reliable predictor of 30-day mortality in both groups. The optimal cutoff score for predicting complications was determined to be 10. The curves indicate that ESS is a useful tool for mortality prediction, particularly in the MENA population (Fig. 4C).

## Discussion

Our findings demonstrate that ESS in general can be considered an excellent tool in predicting mortality and ICU requirements and much better for anticipating postoperative complications. Furthermore, we discovered that applying ESS to different ethnicities, such as those in MENA or non-MENA regions, did not significantly alter results.

Unlike other surgical scores that rely on other intraoperative evaluations or were not created exclusively for emergency cases, ESS was created especially for emergency patients and was based on the presentation of the patient and the presentation of data variables [8–12].

Emergency surgeries require challenging medical care, with mortality and morbidity at higher risk. For instance, the risk of death from EGS is eight times higher than that of elective procedures [1]. Regarding the intensive care requirement, it requires more intensive medical care, especially ventilatory and renal support [13].

Overall, we discovered a strong correlation between ESS and mortality, with a C-statistic of 0.86 (95% CI: 0.80–0.91). Similar results have been reported in previous studies, including one by Kaafarani et al., which showed a C-statistic of 0.84 (95% CI: 0.82–0.87) for mortality prediction in EL patients [14]. Another study found ESS to be significant in predicting mortality in emergency surgeries, including trauma cases, with a C-statistic of 0.88 (95% CI: 0.83–0.93)0.15 The original purpose of ESS was to predict post-EGS mortality, and multiple studies have evaluated its relationship with ICU admissions and EGS-related morbidities, finding consistent and reliable correlations [15, 16].

In our study, we found ESS to be significantly associated with ICU admissions, with a C-statistic of 0.82. This aligns with findings from a multi-center study where a comparable C-statistic of 0.80 was reported.15 Additionally, Napapron et al. found that ESS accurately predicts ICU requirements, with a C-statistic of 0.904 in a cohort of over 11,000 patients [15].

Furthermore, ESS demonstrated good predictive value for post-operative complications, with a C-statistic of 0.79. Nandan et al. also reported a similar association, with ESS predicting complications like bleeding and the need for ventilatory support, with C-statistics of 0.77 and 0.82, respectively [16]. In our study, ESS showed strong prediction for myocardial infarction (C-statistic: 0.92) and acute kidney injury requiring dialysis (C-statistic: 0.88).

ESS had been validated in certain nationalities, such as the United States, Greece in Europe, and Saudi Arabia in the golf area [14, 17, 18]. We took advantage of the multiethnic nature of the Qatari population as it contains population from MENA area and also has almost the same percentage from non-MENA. We compared these two groups regarding the validity of ESS, and we found it a good valid score with almost equal C statistics (Figs. 2, 3 and 4). The median ESS in both groups was non-significant (7.5 for MENA vs. 7 for non-MENA, *P* = 0.45).

In a bi-institutional study comparing two nationality (Greek and American) EL cases, Christou et al. discovered that ESS was significantly beneficial in both groups with relation to postoperative ICU need, mortality percentage, and morbidities [18].

In the literature, the ASA classification showed overestimation of mortality risk and was considered a subjective test, as it depends on factors that may be interpreted in variable ways according to the physician's opinion [19, 20].

Nandan et al. compare ASA and ESS in predicting postoperative complications, and this study revealed that there is a very narrow difference between them (C statistics: 0.78 for ESS vs. 0.77 for ASA), but he recommended ESS as it is more objective and designed specifically for EGS cases, and there are well defined variables [16]. Conversely, Alburakan et al. found a significant difference between ESS and ASA scores regarding mortalities prediction (C statistics: 0.88 vs. 0.78), postoperative complications (C statistics: 0.82 vs. 0.71), and the need for ICU admissions, which also showed that ESS was more significantly superior (C statistics: 0.85 vs. 0.78) [17]. In this study, we confirm the superiority of the ESS over the ASA score in EL cases, especially for mortality (C statistics: 0.86 vs. 0.78) and the need for ICU admission (C statistics: 0.82 vs. 0.77).

According to our statistical method, we defined certain cutoff scores for each of these study outcomes: for mortality, the cutoff score is 10, for complication prediction, the cutoff score is 6, and for the need for ICU admission, the cutoff score is 8. It is adjusted according to institutional resources and plan of care. In the literature, we could find an ESS score of 7 as a cutoff value for ICU admission [15]. Alburakan et al. reported that ESS of 10 was the cut-off value of mortalities; above it, the mortality rate increased dramatically [17]. 

This study has limitations. To test the differences between MENA and non-MENA patients, a larger sample size for some subgroup findings would have been advantageous.

This study represents a single-center data from Hamad Medical Corporation (HMC), the primary healthcare provider in Qatar, comprising three major hospitals (Hamad General Hospital, Al Khor Hospital, and Al Wakra Hospital). While this covers a substantial portion of emergency surgical care in Qatar, the findings may not be fully generalizable to other healthcare systems with different patient populations or resource availability. Additionally, the study lacks surgeon-level variability analysis, as all procedures were performed within a single healthcare system with potentially similar surgical techniques and decision-making patterns. This may limit the applicability of our findings to institutions with different surgical practices. Retrospective medical record reviews have limitations of data quality. Exploring such limitations could be beneficial for future studies. Additionally, our study's focus on emergency laparotomy patients may limit generalizability, as this represents a higher-risk population compared to those eligible for laparoscopic approaches. While this selection reflects real-world scenarios where laparotomy is often reserved for severe cases, our findings may not apply to centers where laparoscopy is the primary emergency approach.

Nevertheless, the study has many strengths. To our knowledge, it is the first to validate ESS among the MENA region, especially in comparison with the non-MENA population, employing many variables. In addition, it is representative of the population living in Qatar, as our institution receives all cases across the country. We are not aware of any previous study that undertook such an analysis, and we hope it will add to the literature for future studies targeting the MENA region’s population.

## Conclusion

We demonstrated that the ESS score is an accurate and reliable tool for predicting 30-day mortality, postoperative complications, and ICU admission in our emergency general surgery (EGS) population. Its performance remained consistent across both MENA and non-MENA patient groups, supporting its generalizability across diverse populations. Given its objectivity and ease of use, ESS offers a practical advantage over traditional tools like ASA, particularly in emergency settings. It can aid acute care surgeons in risk stratification, support informed preoperative counselling, and serve as a benchmark for evaluating and improving the quality of EGS care.

## Data Availability

No datasets were generated or analysed during the current study.
